# Amelioration of Cognitive and Behavioral Deficits after Traumatic Brain Injury in Coagulation Factor XII Deficient Mice

**DOI:** 10.3390/ijms22094855

**Published:** 2021-05-03

**Authors:** Christian Stetter, Simon Lopez-Caperuchipi, Sarah Hopp-Krämer, Michael Bieber, Christoph Kleinschnitz, Anna-Leena Sirén, Christiane Albert-Weißenberger

**Affiliations:** 1 Department of Neurosurgery, University Hospital of Würzburg, Josef-Schneider-Str. 11, 97080 Würzburg, Germany; stetter_c@ukw.de (C.S.); simonlopez@gmx.de (S.L.-C.); hopp_s@ukw.de (S.H.-K.); christiane.albert-weissenberger@uni-wuerzburg.de (C.A.-W.); 2 Department of Neurology, University Hospital of Würzburg, Josef-Schneider-Str. 11, 97080 Würzburg, Germany; bieber_m@ukw.de (M.B.); christoph.kleinschnitz@uk-essen.de (C.K.); 3 Department of Neurology and Center for Translational and Behavioral Neurosciences (C-TNBS), University Hospital of Essen, Hufelandstr. 55, 45147 Essen, Germany; 4 Institute for Physiology, Department for Neurophysiology, Julius-Maximilians-University Würzburg, Röntgenring 9, 97070 Würzburg, Germany

**Keywords:** closed head injury, contact-kinin system, object recognition memory, IntelliCage, Crespi effect, stress

## Abstract

Based on recent findings that show that depletion of factor XII (FXII) leads to better posttraumatic neurological recovery, we studied the effect of FXII-deficiency on post-traumatic cognitive and behavioral outcomes in female and male mice. In agreement with our previous findings, neurological deficits on day 7 after weight-drop traumatic brain injury (TBI) were significantly reduced in FXII^−/−^ mice compared to wild type (WT) mice. Also, glycoprotein Ib (GPIb)-positive platelet aggregates were more frequent in brain microvasculature of WT than FXII^−/−^ mice 3 months after TBI. Six weeks after TBI, memory for novel object was significantly reduced in both female and male WT but not in FXII^−/−^ mice compared to sham-operated mice. In the setting of automated home-cage monitoring of socially housed mice in IntelliCages, female WT mice but not FXII^−/−^ mice showed decreased exploration and reacted negatively to reward extinction one month after TBI. Since neuroendocrine stress after TBI might contribute to trauma-induced cognitive dysfunction and negative emotional contrast reactions, we measured peripheral corticosterone levels and the ration of heart, lung, and spleen weight to bodyweight. Three months after TBI, plasma corticosterone levels were significantly suppressed in both female and male WT but not in FXII^−/−^ mice, while the relative heart weight increased in males but not in females of both phenotypes when compared to sham-operated mice. Our results indicate that FXII deficiency is associated with efficient post-traumatic behavioral and neuroendocrine recovery.

## 1. Introduction

Traumatic brain injury (TBI) is an established risk factor for brain atrophy and dementia [1,2]. Except for the well-documented pathology of axonal damage and enduring neuroinflammation [3], the cellular and molecular mechanisms leading to lasting functional deficits and ongoing neurodegeneration after TBI remain unclear. Recently interest has focused on mechanisms mediating damage to the neurovascular unit encompassing persistent opening of the blood–brain barrier, vascular inflammation and microvascular dysfunction [4]. The coagulation factor FXII (FXII), with its ability to induce and sustain pathologic platelet clotting and inflammation through activation of the complement and contact system [5,6], could be a major player.

Contact activation of FXII generates activated FXII (FXIIa), which triggers the intrinsic coagulation cascade, activates the complement and cleaves plasma prekallikrein to form kallikrein, thus promoting intravascular thrombosis and cellular inflammation [5,6]. FXIIa is required for pathologic clot formation because FXII knockout mice (FXII^−/−^) are resistant to experimentally induced thrombosis [7] or ischemic brain injury [8]. Mechanistic studies in rodent models of cerebral ischemia and traumatic brain injury show that the genetic deletion of FXII or early pharmacological inhibition of FXIIa protects the brain against injury-induced acute platelet activation, microvascular adhesion molecule expression and intraparenchymal proinflammatory cytokine production, thereby reducing blood–brain-barrier damage, cerebral edema and neuronal cell loss and improving functional neurological recovery [8,9,10,11,12]. Importantly, FXII deficiency in humans or mice is not associated with a bleeding phenotype [5,8,11,13]. In mice with thrombotic occlusion of the middle cerebral artery, FXII deficiency even protected them against tissue plasminogen activator (tPA)-induced hemorrhagic transformation [13]. Interestingly, the potential therapeutic efficacy of FXIIa inhibition may not be restricted to acute brain injury: the deficiency, or pharmacologic blockade, of FXIIa delayed disease onset and led to attenuated disease severity in a mouse model of experimental autoimmune encephalomyelitis by reduced demyelination and neuroinflammation [14]. Furthermore, RNA knockdown of FXII with antisense oligonucleotide treatment has been recently reported to ameliorate brain pathology and cognitive impairment in a mouse model of Alzheimer’s disease [15].

We recently reported that FXII genetic deficiency and early pharmacological inhibition of FXIIa improved motor function, reduced brain lesion volume, and diminished neurodegeneration in the acute phase after a TBI [11]. However, whether FXII plays a role in the more protracted post-traumatic behavioral sequelae has not been investigated thus far. Hence, we set out to study the influence of FXII deficiency on post-traumatic behavioral recovery. For this aim we wanted to mimic a mild diffuse head injury by using the established weight-drop closed head injury mouse model [16,17,18]. Cognitive recovery was evaluated using the novel object recognition test and circadian activity and social behavior were studied in the IntelliCages [19]. In order to find out whether gender-specific differences in post-traumatic recovery [20,21] are influenced by FXII deficiency, behavioral monitoring was performed in separate groups of female and male mice.

## 2. Results

**Figure 1:**
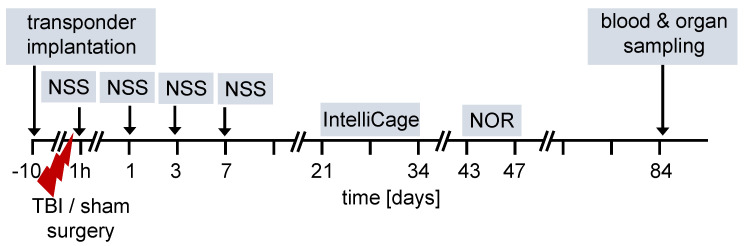
Flow diagram of experimental design. TBI–traumatic brain injury, NSS neuroscore, NOR novel object recognition test.

The overall design of the experiments is shown in the flow chart (Figure 1). We used 12-week old female and male FXII^−/−^ mice and their cage WT littermates for all studies. Ten days before TBI, transponders for identification were implanted under the neck skin. TBI was produced using the weight-drop closed head injury model that produces a diffuse brain trauma [16,17,18]. Trauma severity at 1 h after injury and functional recovery on days 1, 3 and 7 after TBI was tested using a neuroscore (NSS). The time course for behavioral tests and blood and organ sampling are also shown in Figure 1.

### 2.1. Neurological Outcome 7 Days after TBI Is Improved in FXII^−/−^ Mice

**Figure 2:**
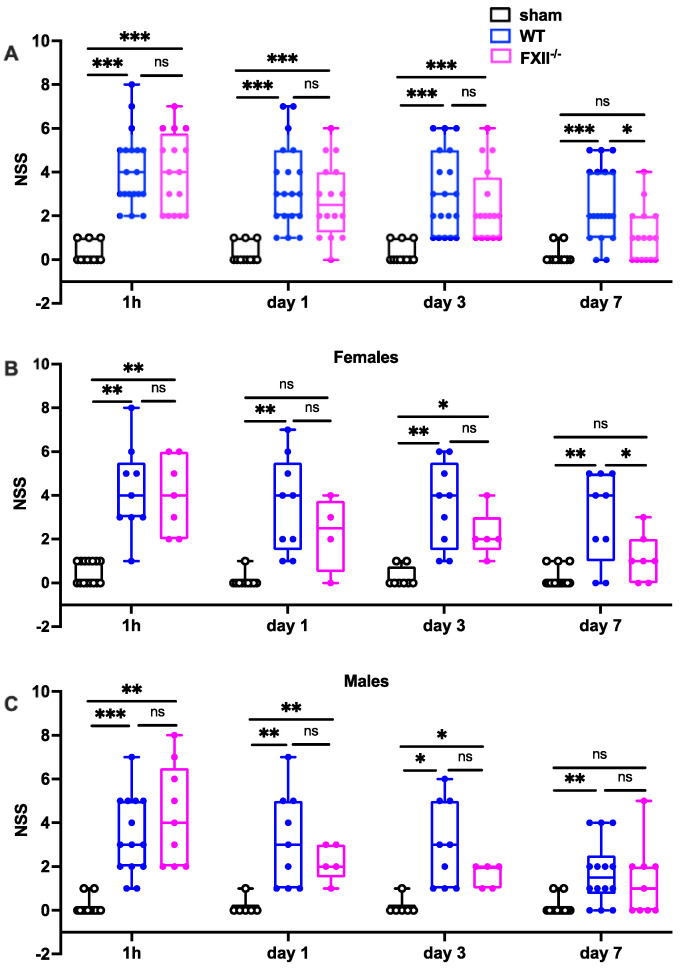
Functional outcome was measured using the Neurological Severity Score (NSS) during the first 7 days following TBI in sham, wildtype (WT) and FXII^−/−^ mice. Neurological deficits at 1 h after TBI and on days 1 and 3 after trauma were similar in FXII^−/−^ and WT mice whereas FXII^−/−^ mice had a significantly better outcome than wild type mice (WT) on day 7 after weight-drop TBI. Number of animals *n* = 25 in sham, *n* = 23 in WT and *n* = 16 in FXII^−/−^ in panel (**A**), in panel (**B**) (females) *n* = 13 in sham, *n* = 9 in WT and *n* = 7 in FXII^−/−^, and in (**C**) (males) *n* = 12 in sham, *n* = 14 in WT and *n* = 9 in FXII^−/−^. Box plots represent median ± 25th and 75th percentiles, whiskers minimum and maximum values, dots indicate individual data points with each boxplot, *** *p* < 0.001, ** *p* < 0.01, * *p* < 0.05, ns = not significant 2-way ANOVA with Bonferroni test.

Trauma severity in the early stages (1 h, 1 and 3 days after TBI) was comparable in all mice, whereas on day 7 after TBI, FXII^−/−^ mice had recovered significantly better than wildtype mice (Figure 2). There were no differences in initial functional trauma severity or functional recovery over the first 7 days between female and male mice (Figure 2).

### 2.2. Microvascular Plated Aggregates Are Still Present 3 Months after TBI in Wildtype Mice

**Figure 3:**
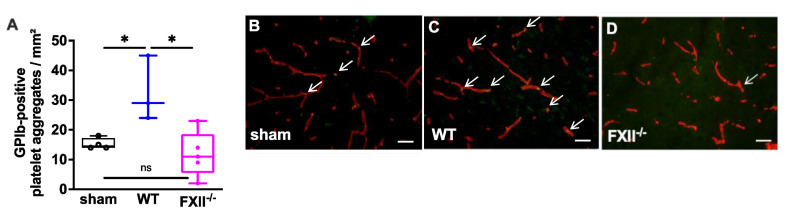
Glycoprotein Ib (GPIb)-positive platelet aggregates in CD31-positive brain microvasculature 3 months after TBI. (**A**). Quantitative analysis of the GPIb immunofluorescence reveals significantly more intravascular platelet depositions in brain sections of wild type mice (*n* = 3) as compared to sham-operated (*n* = 4) or FXII^−/−^ mice (*n* = 5). Box plots represent median ± 25th and 75th per-centiles, whiskers minimum and maximum values, dots indicate individual data points with each boxplot * *p* < 0.05, ns = not significant one-way ANOVA with Bonferroni test. In panels (**B**) to (**D**) images of the frontoparietal cortex showing yellow-green GPIb-positive platelet aggregates (white arrows) in CD31-positive brain microvessels (red) in (**B**) sham, (**C**) wild type (WT) and (**D**) FXII^−/−^ mouse; scale bar = 50 µm.

In a previous study we found microvascular platelet aggregates in the brain tissue of wild type mice 1 week after TBI as well as in the brain parenchyma of patients with traumatic brain injury [11]. To find out whether the TBI-induced intracerebral thrombus formation was still detectable after trauma, we analyzed brain tissue 3 months after TBI. As shown in Figure 3 intravascular accumulations of GPIb-positive platelets were found in the cortical brain tissue of 3 wild type, 5 FXII^−/−^ and 4 sham mice. The number of platelet aggregates in wild type mice was significantly more when compared with the brain sections of FXII^−/−^ or sham mice.

### 2.3. FXII Deficiency Is Associated with Better Behavioral Outcome after TBI

**Figure 4:**
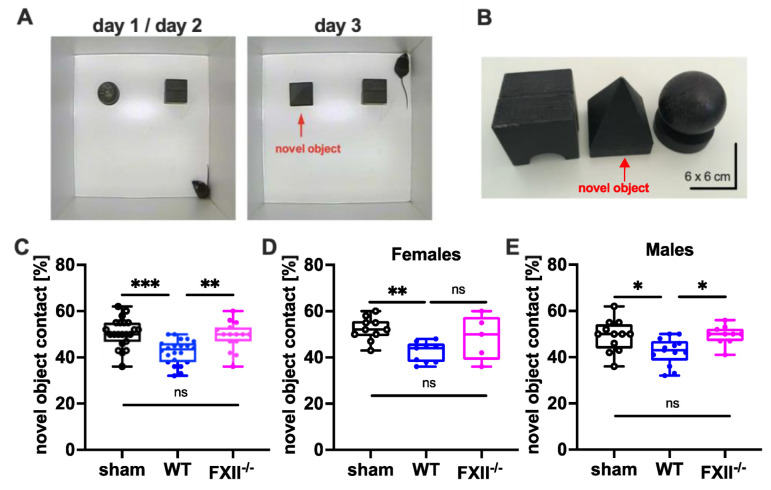
Cognitive recovery in novel object recognition test in sham (*n* = 22), wild type (WT, *n* = 22) and FXII^−/−^ mice (*n* = 15). (**A**) Setup for the novel object test on days d1/d2 and d3. (**B**) Black wooden objects of 6 × 6 × 6 cm size were used in the test trials. The novel object used on day 3 is marked with a red arrow. (**C**–**E**) Retention memory for novel object 6 weeks after TBI was significantly reduced in wild type mice as compared to sham-operated or FXII^−/−^ mice. Number of animals *n* = 22 in sham, *n* = 22 in WT and *n* = 15 in FXII^−/−^ in panel (**C**), *n* = 10 in sham, *n* = 9 in WT and *n* = 5 in FXII^−/−^ in panel (**D**) (females), and *n* = 12 in sham, *n* = 13 in WT and *n* = 10 in FXII^−/−^ in panel (**E**) (males), *** *p* < 0.001,** *p* < 0.01, * *p* < 0.05, ns = not significant one-way ANOVA with Bonferroni test.

To test cognitive recovery after TBI we used the object recognition test [22]. The trial setup and objects used are shown in Figure 4A,B. During training on days 1 and 2, the mice were allowed to explore 2 test objects for 10 min. During recall on day 3, one of the objects (ball-shaped) was replaced with a novel one (pyramid-shape). The novel object recognition in wild type mice was impaired when compared to sham-operated or FXII-deficient mice (Figure 4C). The memory deficit was evident in both female and male wild type mice compared to sham-operated mice (Figure 4D,E).

**Figure 5:**
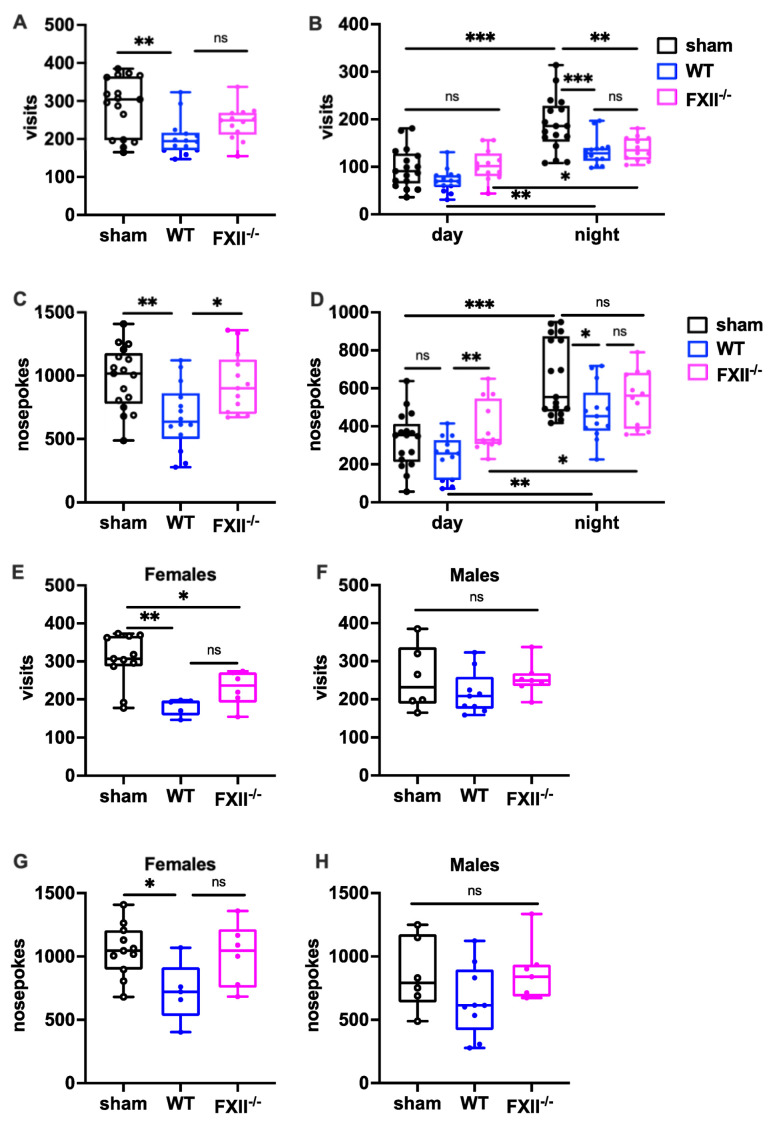
Behavioral phenotyping after TBI in sham, wildtype (WT) and FXII^−/−^ mice. Investigative activity reflected by visits (**B**–**D**) and nosepokes (**E**–**G**) in the IntelliCage corners during the first day of testing is significantly lower in wild type mice than in sham-operated or FXII^−/−^ mice 3 weeks after TBI. All mice made more visits and nosepokes during dark night period (7 pm to 7 am) than during daytime (7 am to 7 pm) when light was on. Number of animals *n* = 17 in sham, *n* = 14 in WT and *n* = 13 in FXII^−/−^ in panels (**A**–**D**), in panels (**E**) and (**G**) (females) *n* = 11 in sham, *n* = 5 in WT and *n* = 6 in FXII^−/−^, and in (**F**) and (**H**) (males) *n* = 6 in sham, *n* = 9 in WT and *n* = 7 in FXII^−/−^. Box plots represent median ± 25th and 75th per-centiles, whiskers minimum and maximum values, dots indicate individual data points with each boxplot, *** *p* < 0.001, ** *p* < 0.01, * *p* < 0.05, ns not significant one-way ANOVA with Bonferroni test).

To measure the behavioral consequences of TBI, we used the IntelliCage system, which allows fully automated testing of spontaneous activity and behavior in group-housed mice. When exploratory activity was monitored during a 24 h period, wild type mice, but not FXII-deficient mice subjected to weight-drop injury, showed corner visits and nosepokes (Figure 5A,C). In general, all mice made more corner visits and nosepokes during the dark phase compared to the light phase (Figure 5B,D) The TBI-induced reduction in exploration was also seen more clearly during the active dark phase (Figure 5B,D). Stratification by gender revealed that both the wildtype and FXII^−/−^ females made fewer corner visits, and wildtype females fewer nosepokes than sham-operated female mice (Figure 5E,F), whereas no significant differences were found in the exploratory activity of male mice (Figure 5G,H).

**Figure 6:**
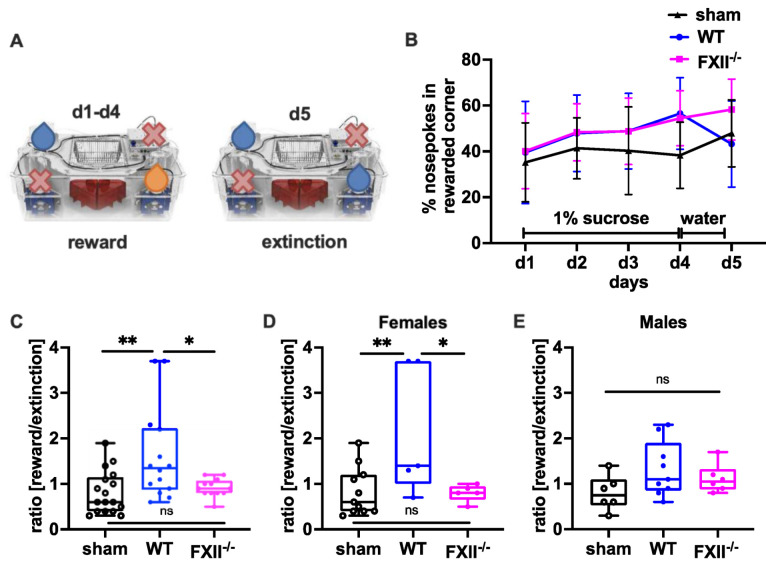
Behavioral phenotyping after TBI in sham, wild type (WT) and FXII^−/−^ mice. (**A**) Setup for appetitive reward and extinction in IntelliCages. During the trial water or 1% (*w*/*v*) sucrose water was available at designated corners from 10 p.m. to 12 p.m. Blue droplets depict corners with access to water, orange droplets access to 1% (*w*/*v*) sucrose water, and red crosses closed corners with no drinking access. (**B**) Percent of nosepokes in the rewarded corner during the reward/extinction testing. (**C**–**E**) Negative emotional response to appetitive reward extinction 4 weeks after TBI: Wild type mice in contrast to FXII^−/−^ or sham-treated mice avoid using the previously rewarded corner when the drinking supply is shifted from 1% sucrose to water on day 5 of testing. ** *p* < 0.01, * *p* < 0.05, ns no significant difference, one-way ANOVA with Bonferroni test, Number of animals *n* = 17 in sham, *n* = 14 in WT and *n* = 13 in FXII^−/−^ in panel (**C**), *n* = 11 in sham, *n* = 5 in WT and *n* = 6 in FXII^−/−^ in panel (**D**) (females) and *n* = 6 in sham, *n* = 9 in WT and *n* = 7 in FXII^−/−^ in panel (**E**) (males). Line graphs represent mean ± SD, box plots median ± 25th and 75th percentiles, whiskers minimum and maximum values, dots indicate individual data points with each boxplot, ** *p* < 0.01, * *p* < 0.05, ns = not significant one-way ANOVA with Bonferroni test.

In a set-up aimed to assess the negative contrast effect after reward withdrawal [23,24,25], we monitored sucrose preference by comparing the percentage of nosepokes in the sucrose-rewarded corner before and after replacement of the sucrose reward with water (Figure 6A). Even if the overall sensitivity towards reward gain during the sucrose consumption phase on days 1–3 was not was significantly different between the groups (Figure 6B), both wild type and FXII^−/−^ mice made significantly more nosepokes at the sucrose rewarded corner on day 4 compared to sham-operated mice (mean ± SD sham 38 ± 14%, *n* = 17, wildtype 57 ± 15%, *n* = 14, *p* = 0.002 in wild type and FXII^−/−^ 53 ± 12%, *n* = 12, *p* = 0.012 one-way ANOVA with Bonferroni test). On day 5, when the sucrose water was replaced with non-sweetened water, wild type but not FXII^−/−^ mice showed a strong negative contrast effect (Figure 6C). When the data was stratified for gender, only wild type females showed increased preference to the sucrose rewarded corner (mean ± SD 59 ± 19%, *n* = 5 in WT females vs. 36 ± 16%, *n* = 11 in sham females, *p* = 0.018, one-way ANOVA with Bonferroni test) and reacted negatively to reward extinction (Figure 5E and Figure 6D).

### 2.4. Plasma Corticosterone Is Reduced in Wild Type Mice 3 Months after TBI

**Figure 7:**
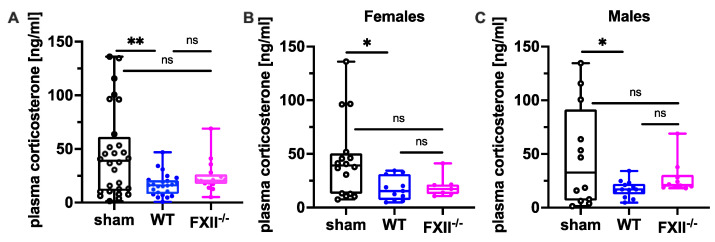
Plasma corticosterone level 3 months after TBI in sham, wild type (WT) and FXII^−/−^ mice. Peripheral corticosterone levels are decreased in wild type mice, but not in FXII^−/−^ mice when compared to sham-operated mice. Number of animals *n* = 25 in sham, *n* = 23 in WT and *n* = 16 in FXII^−/−^ in (**A**), *n* = 13 in sham, *n* = 9 in WT and *n* = 7 in FXII^−/−^ in (**B**) (females) and *n* = 12 in sham, *n* = 14 in WT and *n* = 9 in FXII^−/−^ in (**C**) (males). Box plots represent median ± 25th and 75th percentiles, whiskers minimum and maximum values, dots indicate individual data points with each boxplot, ** *p* < 0.01, * *p* < 0.05, ns = not significant one-way ANOVA with Bonferroni test.

Since suppression of the neuroendocrine-immune system after TBI might contribute to cognitive dysfunction, negative emotional contrast reactions, and reduced exploratory activity (Figure 4 and Figure 5), we measured peripheral corticosterone levels at the sub-chronic phase after TBI. Plasma corticosterone levels were significantly depressed 3 months after TBI in both female and male wild type mice compared to sham-operated mice, whereas in FXII^−/−^ mice, plasma corticosterone levels were not different from sham-operated mice (Figure 7).

### 2.5. Heart Weight to Body Weight Ratio Is Increased in Male Mice 3 Months after TBI

Heart weight to body weight ratio was significantly increased 3 months after TBI in both wild type and FXII^−/−^ males when compared to sham-operated mice whereas in female mice, the ratio was not different from sham-operated mice (Table 1). No significant differences were found in lung or spleen weights between the groups (Table 1).

## 3. Discussion

In the present study, FXII-deficiency was associated with a better post-traumatic cognitive and behavioral outcome and neuroendocrine stress-response in mice. Post-traumatic deficits in retention memory were prominent 1 month after head injury in wild type mice whereas FXII deficient mice exhibited complete post-traumatic recovery. We also found that traumatized wild type mice were more sensitive to reward loss than FXII-deficient mice although their sensitivity towards reward gain remained unaffected. The negative contrast represents an established behavioral response in rodents to reward extinction [23,24,25]. Anatomically negative contrast behavior is dependent on the amygdala rather than on memory-relevant brain areas, such as the hippocampus [26,27]. Interestingly, the TBI-induced negative contrast was seen in female but not in male mice. It is unlikely that the reduced nosepokes in the extinguished corner represented learning since cognitive defects were equally impaired after TBI in both female and male mice. The more sustained “disappointment” of traumatized female mice suggested that they were less resilient towards negative emotions. They behaved similarly to mice that experienced affective states like depression and anxiety [28]. This finding was interesting in view of the fact that post-TBI women show more severe post-concussion symptoms, depression, and anxiety [21].

Though the mechanism for the apt neuropsychological recovery in FXII-deficient mice is not fully clear, the lack of a profound FXII-dependent thrombo-inflammation after brain injury [8,11,12,29] may have contributed to this behavioral resilience. This possibility gains support from research that found that microthrombi were abundantly present in cerebral vessels surrounding brain lesions 3 months after weight-drop TBI in wildtype mice whereas no augmented formation of platelet deposits was found in brain tissue of FXII-deficient mice. Interestingly, depletion of FXII was recently reported to ameliorate brain pathology and cognitive impairment in a mouse model of Alzheimer’s disease [15]. Similar to activation of the contact kinin system in human head injury [30] and in mouse models of TBI [11,12,31], the activation of FXII and other kallikrein–kinin system components was reported in the plasma of Alzheimer’s patients and in a mouse model of Alzheimer’s disease [32]. The authors proposed that a beta-amyloid-mediated activation of FXII in Alzheimer’s disease gives rise to the formation of microthrombi and inflammation and contributes to cognitive decline [32,33,34].

TBI is heterogeneous and the behavioral consequences of TBI may depend on induced injury patterns and injury severity [18,35]. In contrast to a recent study investigating behavior using IntelliCages in mice after controlled cortical impact (CCI) with profound focal injury [36], we did not observe hyperactivity in the IntelliCages in the present study. This may be due to differences in the injury model and severity [11,16,37,38]. In our mouse model, post-traumatic cognitive dysfunction was seen in both female and male wild type mice, whereas the negative emotional contrast reaction and reduced exploratory activity were particularly prominent in female wild type mice. Similar to cognitive impairment, reduced plasma corticosterone levels 3 months after weight-drop brain injury were detected in both female and male wild type mice compared to sham mice, whereas in FXII-deficient mice, the circulating levels of corticosterone were not significantly different from those of sham-operated mice. Dysfunction of the hypothalamic–pituitary–adrenal (HPA) axis after human and experimental TBI is common [39,40] and likely contributes to post-traumatic neurocognitive impairments [41]. Resting levels of plasma corticosterone were decreased, and stress-induced corticosterone responses were blunted 2 months after a midline fluid percussion injury in rats [42]. Chronic suppression of the endocrine stress response was also reported in rats 10 months after controlled cortical impact injury [43] and low basal morning levels of cortisol were reported in 25–45% of patients 1 year after TBI [44,45]. Stress-induced neurohormonal changes can lead to acute cardiac dysfunction after severe brain damage after stroke and TBI [46,47], resulting in chronic cardiac dysfunction and hypertrophy [48,49,50]. We found cardiac hypertrophy 3 months after TBI in both wild type and FXII^−/−^ male mice. Similar to our present findings, an increased heart weight-to-bodyweight ratio was reported 30 days after controlled cortical impact in male mice [49]. Interestingly post-traumatic cardiac hypertrophy was associated with a strong immune cell response but was prevented by a splenectomy immediately before injury [49]. A splenectomy, however, had no effect on neurocognitive deficits [49]. In the present study FXII-deficiency prevented post-traumatic memory loss but had no effect on heart or spleen weight.

One limitation of this study is that the FXII deletion was studied in a genetic mouse model. While we have shown before that therapeutic inhibition of the FXIIa leads to a better outcome in the acute phase [11], it is however, unknown whether this also has a chronic lasting effect. Future studies with therapeutic inhibition of FXIIa in wild type mice could strengthen the concept for a clinical translation.

In conclusion, FXII deficiency is associated with efficient post-traumatic behavioral and neuroendocrine recovery. These novel findings add to the well-characterized pathological actions of FXII on clotting and inflammation mediated by the contact kinin system [5,11,12,51]. Thus, inhibition of FXII activation could be a future therapeutic option to improve post-traumatic neurocognitive recovery, especially since it can be achieved without increasing the risk of bleeding [5,8,11].

## 4. Materials and Methods

### 4.1. Animals

A total of 33 (18 male, 15 female) 12-week old Factor XII deficient (FXII^−/−^) mice on C57BL66N background [7,8] and 37 (20 male, 17 female) wildtype (FXII^+/+^) littermates were used in this study. Ten days before head injury, the mice were anesthetized with 2% isoflurane in 100% oxygen and an ISO micro transponder (8.5 × 1.2 mm, PM 162-8, TSE Bad Homburg, Germany) was implanted below the skin of the neck using a specific device (transponder injector, TSE Bad Homburg, Germany). During the experiments, the mice were housed in groups of 14 to 16 in standard 20.5 × 55 × 38.5 cm plastic cages (height × length × width, Tecniplast, 2000 P, Techniplast Deutschland, Hohenpeißenberg, Germany), with food (standard mouse pellets) and water ad libitum, a temperature maintained at 20–22 °C, and a 12 h light–dark cycle (lights on at 7:00 a.m.).

### 4.2. Weight-Drop Close Head Injury

A closed-head traumatic brain injury was produced as previously described [16,38]. Briefly, the mice were anesthetized and maintained with 2% isoflurane anesthesia in 100% oxygen during the whole procedure. A midline longitudinal scalp incision was made to expose the skull. The head was fixed by holding it with two fingers to keep the mice in the right position and to allow a slight movement at the moment of the trauma induction. After identification of the impact area over the right fronto–parietal cortex, TBI was induced by a falling weight (95 g) with a silicone-covered blunt tip onto the skull with a final impact of 0.01 J. After TBI induction, the mice shortly received 100% oxygen. The skull was examined to preclude fractures and the skin closed. Sham operation included anesthesia and exposure of the skull, but without weight-drop injury. The neurobehavioral status of the mice was assessed by the neurological severity score (NSS) [16,17], a composite score including tasks on motor function, alertness, and physiological behavior, with higher scores indicating a more severe deficit. Functional testing was performed initially after 1 h, and repeated on day 1, 3 and 7 after weight-drop injury by investigators blinded to the experimental groups.

### 4.3. Evaluation of Post-Traumatic Cognition, Anxiety-Related, and Sensorimotor Behavior

#### 4.3.1. Object Recognition Test

An object recognition task involving memory of a familiar object in parallel with the detection and encoding of a novel object was performed. This test was based on the spontaneous tendency of mice to spend more time exploring a novel object than a familiar one and also to recognize when an object was relocated [22]. The setup and type of objects used are shown in Figure 4A,B. During a two-day initial habituation phase a mouse was placed in the middle of the test arena (40 × 40 × 40 cm) and was allowed to explore the empty arena for 10 min. During day 1 and 2 of training trials, the mice were allowed to explore two wooden black painted objects (6 × 6 × 6 cm) that had been placed in the arena. A mouse was allowed to explore the arena for 10 min. On day 3, one object was replaced by a novel object of similar material but of different shape (Figure 4). The order of the tests was randomized except for the fact that we always tested animals from one cage in a row. The arena and the objects were wiped clean with 70% ethanol between each trial to remove any traces of urine or feces of an individual mouse from the same cage. The test arena and the objects were thoroughly cleaned first with water and then with 70% ethanol between trials of mice from different cages to preclude odor recognition. Exploration of an object was defined as goal-oriented sniffling or touching with nose, forepaw or vibrissae. A camera mounted above the arena recorded the number of contacts the mouse made by actively sniffling the object.

#### 4.3.2. IntelliCage Test

The automated testing chamber IntelliCage system (IntelliCage New Behavior, TSE Systems GmbH, Bad Homburg, Germany) is designed to monitor automated home-cage behavior of mice in groups with a minimum of human handling [19,24]. The system is set into a large mouse cage (20 × 55 × 38 cm, Tecniplast, 2000 P) covered by standard bedding material. Male and female mice were always housed in separate cages, and sham, WT and FXII^−/−^ mice were group-housed together. Four triangular, red mouse houses (Tecniplast^®^) in the middle of the cage serve as platforms for reaching food, sleeping quarters and environmental enrichment to minimize aggressive male cage behavior. The four operant corners were each equipped with two water bottles with access controlled by sensors and doors. The corners were integrated with radio frequency antennas to allow selective door opening for each mouse equipped with an ISO transponder when entering the corner. To access water, the mouse had to make a nosepoke to open the doors. The set-up was controlled by special software that can be programmed for experimental tasks and schedules. The number and duration of corner visits, nosepokes, and licks were automatically recorded without the need to handle the mice. The test paradigm depicted in Figure 6A includes test phases for sucrose preference, reward extinction and negative emotional contrast reaction (the “Crespi” effect) when the mice were able to drink ad libitum sucrose water that was subsequently withdrawn [23,24]. In the setup, sucrose preference was monitored by comparing the percentage of nosepokes in the sucrose-reward corner before and after replacement by water (Figure 6A). During the trial, water or 1% (*w*/*v*) sucrose water was available in designated corners from 10 p.m. to 12 p.m. on days 1 to 4. It was replaced with unsweetened water on day 5.

### 4.4. Organ Weight to Body Weight Ratio

At the end of the functional testing, bodyweight was determined and after sacrifice by decapitation under deep fluothane anesthesia, the organs were immediately excised, washed with 1xPBS, dry blotted, weighed and the ratios of heart, lung and spleen weight to bodyweight were determined [50].

### 4.5. Immunohistochemistry

Cryo-embedded mouse brains were cut into 15-μm slices using a cryostat (Leica Biosystems, Wetzlar, Germany). For immunofluorescence staining, cryosections were fixed with 4% paraformaldehyde (PFA, Sigma-Aldrich Merck, Darmstadt, Germany) in phosphate buffered saline (PBS, Sigma) for 15 min at room temperature (RT), rinsed 2 × 10 min with PBS and stained with the following primary antibodies: rat monoclonal anti-mouse glycoprotein Ib (GPIb) (1:100; emfret ANALYTICS, Eibelstadt, Germany), and rat monoclonal anti-mouse CD31 (MCA2388GA, 1:100; Bio-Rad Laboratories, Hercules, CA, USA). As secondary antibodies, Cy2 anti-rat (122-225-167, 1:100; Dianova, Hamburg, Germany) and Cy3 anti-rat (712-165-150, 1:100; Dianova) were used. Images of the immunofluorescence staining were acquired using a Nikon Eclipse 50i microscope equipped with Digital Sight camera (Nikon, Tokyo, Japan) and processed further with NIS-Elements imaging program (Nikon). GPIb-positive platelet aggregates in CD31-positive intraparenchymal microvessels were counted on five microscopic fields of the perilesional frontoparietal cortex in two brain slices per animal in a blinded fashion.

### 4.6. Corticosterone Assay

Plasma samples were collected from the retro-orbital venous plexus under isofluorane inhalation (2% in O_2_) before sacrifice at 3 months after TBI and stored at −80 °C until the determination of corticosterone levels with a commercially available mouse/rat corticosterone ELISA kit (AR E-8100, LDN Labor Diagnostika Nord GmbH, Nordhorn, Germany) following the manufacturer’s protocol. Each sample was run in duplicate. Ten microliters of each sample, standard and control were added into the wells followed by 100 µL of incubation buffer and 50 µL of enzyme conjugate. After 2 h incubation on a shaker at room temperature, the microplates were washed four times, and 200 µL of substrate solution was added. The plates were then incubated for 30 min in the dark at room temperature and the reaction stopped by adding 50 µL of stop solution. The absorbance was determined at 450 nm on a LabSystems Multiskan EX microplate reader (Thermo-Scientific, Regensburg, Germany). The concentration of corticosterone was inversely proportional to the optical density measured in a microplate reader. The sensitivity of the assay was 6.1 ng/mL.

### 4.7. Statistical Analysis

The number of animals necessary to detect a Crespi effect (see above) after sugar water withdrawal in the IntelliCage system was determined via an a priori sample size calculation with the following assumptions: α = 0.05, β = 0.2, mean, and standard deviation (G*Power 3.0.10, [52]). To avoid bias, experiments were performed and analyzed in a blind fashion. The results were expressed as mean ± SD or as box-and-whisker plots (horizontal line median, boxes 25th and 75th, whiskers 10th and 90th percentile). For statistical analysis PrismGraph 9.1.10 software package (GraphPad Software, GraphPad Inc, La Jolla, CA, USA) was used. Data were tested for Gaussian distribution with the Kolmogorov–Smirnov and Shapiro–Wilks test. The group differences of neuroscores were analyzed using two-way repeated ANOVA measures with a post hoc Bonferroni test. All other data comparisons were performed using one-way ANOVA with post hoc Bonferroni correction.

## Figures and Tables

**Table 1:** Organ weight to body weight (OW/BW) ratios for heart, lung and spleen 3 months after TBI in sham-operated, wild type (WT) and FXII^−/−^ mice. Relative heart weights were increased in both wild type and FXII^−/−^ male mice after TBI when compared to sham-operated males (** *p* < 0.01, * *p* < 0.05, one-way ANOVA with Bonferroni test). Data represent mean (±SD) and median (±25th and 75th percentiles) for the given number of animals (*n*).

OW/BW (mg/g)	Male			Female		
	Sham (*n* = 10)	WT (*n* = 8)	FXII^−/−^ (*n* = 9)	Sham (*n* = 13)	WT (*n* = 9)	FXII^−/−^ (*n* = 6)
**Heart**						
mean (±SD)	4.64 (±0.73)	5.42 (±0.42) *	5.70 (±0.60) **	4.75 (±1.16)	5.34 (±0.59)	5.14 (±1.06)
median	4.48	5.43	5.92	4.35	5.3	5.12
(± 25%,75%)	(±4.03, 5.14)	(±5.06, 6.18)	(±5.14, 6.15)	(±3.85, 5.76)	(±5.03, 5.76)	(±4.08, 6.13)
**Lung**						
mean (±SD)	8.03 (±1.19)	7.63 (±0.55)	8.36 (±1.30)	11.0 (±1.06)	10.5 (±1.39)	9.70 (±2.07)
median	7.74	7.38	8.38	11.3	10.2	10.3
(± 25%,75%)	(±7.04, 9.13)	(±7.29, 8.21)	(±7.46, 8.75)	(±10.1, 11.8)	(±9.25, 11.6)	(±7.28, 11.4)
**Spleen**						
mean (±SD)	3.66 (±0.62)	3.69 (±0.50)	4.11 (±0.79)	4.97 ± (0.89)	4.27 (±0.61)	4.42 (±0.74)
median	3.59	4	3.91	5.17	4.19	4.16
(± 25%,75%)	(±3.18, 4.26)	(±3.10, 4.06)	(±3.64, 4.29)	(±4.16, 5.45)	(±3.88, 4.75)	(±3.82, 5.20)

## Data Availability

The data presented in this study are available on request from the corresponding author. The data are not publicly available at this point due to intellectual property/confidentiality issues.
